# Spoken language processing activates the primary visual cortex

**DOI:** 10.1371/journal.pone.0289671

**Published:** 2023-08-11

**Authors:** Anna Seydell-Greenwald, Xiaoying Wang, Elissa L. Newport, Yanchao Bi, Ella Striem-Amit

**Affiliations:** 1 Center for Brain Plasticity and Recovery, Georgetown University Medical Center, Washington, DC, United States of America; 2 State Key Laboratory of Cognitive Neuroscience and Learning & IDG/McGovern Institute for Brain Research, Beijing Normal University, Beijing, China; 3 Department of Neuroscience, Georgetown University Medical Center, Washington, DC, United States of America; Museo Storico della Fisica e Centro Studi e Ricerche Enrico Fermi, ITALY

## Abstract

Primary visual cortex (V1) is generally thought of as a low-level sensory area that primarily processes basic visual features. Although there is evidence for multisensory effects on its activity, these are typically found for the processing of simple sounds and their properties, for example spatially or temporally-congruent simple sounds. However, in congenitally blind individuals, V1 is involved in language processing, with no evidence of major changes in anatomical connectivity that could explain this seemingly drastic functional change. This is at odds with current accounts of neural plasticity, which emphasize the role of connectivity and conserved function in determining a neural tissue’s role even after atypical early experiences. To reconcile what appears to be unprecedented functional reorganization with known accounts of plasticity limitations, we tested whether V1’s multisensory roles include responses to spoken language in sighted individuals. Using fMRI, we found that V1 in normally sighted individuals was indeed activated by comprehensible spoken sentences as compared to an incomprehensible reversed speech control condition, and more strongly so in the left compared to the right hemisphere. Activation in V1 for language was also significant and comparable for abstract and concrete words, suggesting it was not driven by visual imagery. Last, this activation did not stem from increased attention to the auditory onset of words, nor was it correlated with attentional arousal ratings, making general attention accounts an unlikely explanation. Together these findings suggest that V1 responds to spoken language even in sighted individuals, reflecting the binding of multisensory high-level signals, potentially to predict visual input. This capability might be the basis for the strong V1 language activation observed in people born blind, re-affirming the notion that plasticity is guided by pre-existing connectivity and abilities in the typically developed brain.

## Introduction

What are the multisensory or cognitive inputs to primary sensory cortices? Despite the canonical view of the primary visual cortex (V1) as a unisensory, low-level processing station, recent decades have provided ample evidence for multisensory integration in the early visual cortex [1–3]. Studies have convincingly shown early visual cortex responses to, modulation of, or causal involvement in the processing of simple sound stimuli (e.g. [4–10]; see [1] for a review.) Usually, these V1 responses are based on low-level features of the auditory stimuli, such as spatial location or temporal synchrony with visual stimuli, allowing for efficient visual responses [11–13]. It is less clear whether the early visual cortex also receives high-level information pertaining to object category, congruence, or language. While one study reported that V1 signal patterns allow discrimination based on the categorical content of sounds [14], several other studies did not find such high-level modulations [15–22].

There is one case, however, where the early visual cortex is known without a doubt to respond to higher-level non-visual processing: the primary visual cortex (V1) of people born blind has been shown to be involved in language comprehension and production [23–29], and its involvement in language is often left-lateralized [23, 24, 26, 30–35] like the typical fronto-temporal language activation observed in many neuroimaging studies of sighted people (for reviews, see [36, 37]). This apparent role of V1 in language processing in people born blind appears to be a marked deviation from its function in sighted individuals, where V1 is involved in the processing of basic visual features [38–41]. Although evidence for V1 language activation in congenitally blind people is compelling, persuasive evidence for a mechanism by which such extreme functional change from low-level visual to language processing might occur has not been provided to date. Beyond deterioration of the visual pathways [42–45], no drastic differences in anatomical connectivity of the visual cortex have been found between congenitally blind people and sighted controls. Importantly, most recent research suggests that brain organization is strongly determined by anatomical connectivity present already at birth [46–49]. This view implies that functional reorganization needs to build on, and is limited by, pre-existing capacities and connections of the available tissue, even in cases of sensory deprivation since birth [50, 51]. How can findings of language processing in primary visual cortex in the congenitally blind be reconciled with such a view? If the hypothesis of pre-existing anatomical connectivity and its constraints is correct, then to accord with language processing in primary visual cortex in the blind, there must also be language processing, or related inputs, in primary visual cortex in sighted people.

Here we tested whether language processing recruits V1 in sighted adults, with the dual goal of testing cognitive engagement of early sensory cortices, and addressing the roots of plasticity in blindness. We report a series of experiments that demonstrates V1 activation by spoken language while addressing potential confounds of visual imagery and attention. Experiment 1 employs a robust auditory sentence comprehension task, as compared with a low-level control (backward speech, which is not comprehensible), in 20 neurologically healthy sighted young adults, and Experiments 2 and 3 examine the potential influence of visual imagery in an independent second cohort by testing responses to auditorily presented abstract words, which are hard to visualize. If language indeed activates primary visual cortex during sentence processing in a typically-developed cohort, such activation may be the basis for the more extreme, previously unaccounted for, plasticity in blindness. Further, in the sighted brain, it would support the notion of cognitive responsivity in the primary visual cortex.

## Materials and methods

### Participants

#### Experiment 1

Participants were 20 young adults (5 men, ages 18 to 38, mean 21.8 years) with normal or corrected-to-normal vision and no history of neurological disorder from the Georgetown University community. All were native speakers of English and had not been fluent in any other language by the age of 12. All experimental protocols were approved by the institutional review board of Georgetown University Medical Center, in accordance with the Declaration of Helsinki. Participants provided informed consent and were compensated for their time.

#### Experiments 2, 3

Participants were 14 adults with normal or corrected-to-normal vision and no history of neurological disorder (8 men, ages 23 to 66, mean 43.85 years). All were native speakers of Mandarin Chinese. All experimental protocols were approved by the institutional review board of the Department of Psychology at Peking University, China, as well as by the institutional review board of Harvard University, in accordance with the Declaration of Helsinki. Participants provided informed consent and were compensated for their time.

### Experimental design

#### Experiment 1

The fMRI language task used here was a modified version of an Auditory Description Decision Task used to determine language dominance prior to epilepsy surgery [52, 53]. In the Forward Speech condition, participants heard short English sentences (e.g., “A big gray animal is an elephant”, “Birthday cake lights are candles”, “Something that reflects your image is a beaver”) and pushed a button if they considered the sentence a true statement. In the Reverse Speech condition, they heard the same sentences played in reverse (thus rendered incomprehensible) and pushed a button when they heard a soft beep inserted at the end of the utterance. Audio files with example stimuli can be found in the **S1–S4 Audios**. The proportion of correct statements and reverse speech utterances with beeps was 50%. The task was designed to be easy; performance was nearly perfect (median performance at 100% for both tasks, mean 97.2±4.5% for sentence comprehension, mean 99.5±1.1% for beep detection). Each participant completed two fMRI runs of 5 min and 48 s duration, each containing four 30-second blocks of each of two experimental conditions (Forward and Reversed Speech, six utterances per block) in counterbalanced order, with 12-second silent rest periods at the beginning and end of the run, as well as in between each of the eight active blocks. Aside from a fixation cross that participants were asked to rest their eyes on throughout the scan, no visual stimulation was provided. The Forward>Reverse activation differences evoked by this task are highly robust and reproducible, making them suitable for localizing language-associated brain areas across development [54] and even in cases of atypical functional organization, such as participants with a history of chronic epilepsy [53] or perinatal stroke [55]. Importantly, the sentences were spoken with neutral prosody, so that potential modulation of V1 activation by emotional auditory stimuli [56] is unlikely.

Imaging data were acquired on Georgetown’s research-dedicated 3T Siemens Trio Tim scanner with a 12-channel birdcage head coil. Auditory stimuli were delivered via insert earphones (Sensimetrics S14) worn under ear defenders (Bilsom Thunder T1). Stimulus presentation and response collection (via a Cedrus fiber optics button box) were coordinated by E-Prime 2.0 software. Each of the two functional runs contained 100 functional (T2*-weighted) volumes covering the whole brain in 50 slices acquired in descending order and oriented parallel to the AC-PC plane (EPI parameters: TE = 30 ms, TR = 3000 ms, flip angle = 90°, matrix 64 × 64, slice thickness = 2.8 mm, distance factor = 7%, resulting in an effective voxel size of 3 × 3 × 3 mm). A high-resolution anatomical (T1-weighted) scan was acquired for co-registration (MPRAGE parameters: whole-brain coverage in 176 sagittal slices, TE = 3.5 ms, TR = 2530 ms, TI = 1100 ms, flip angle = 7°, matrix 256 × 256, voxel size = 1 × 1 × 1 mm).

#### Experiments 2, 3

Stimuli in these experiments were spoken words, each a two-character word in Mandarin Chinese, belonging to eight categories: abstract concepts (e.g. “freedom”, “truth”, “wish”), concrete everyday object names (e.g. “cup”, “closet”, “computer”), and six additional categories which were not analyzed in the current manuscript (astral/weather phenomena—e.g. “rainbow”, “rain”; scenes—“island”, “beach”; and object features—colors and shapes, e.g. “red”, “square”; see full detail in [57]). Each category included ten words whose imaginability and attentional arousal (as well as other measures not used here) were rated on a 7-point scale [58] by an independent sample of 45 sighted Chinese participants with similar levels of education. The concrete objects and abstract concepts compared in **Fig 2B** differed significantly in imaginability (Welch t-test contrast, p < 0.001, significant after Bonferroni correction for multiple comparisons including other measures not used here; [57]), but not quite in arousal (p = 0.06 uncorrected; does not surpass the corrected threshold for multiple comparisons).) During both experiments, participants kept their eyes closed and responded with a button push to rare catch trials (occurrences of a fruit name among the other words.) Runs with more than one missed catch trial were excluded from the analysis, as were the imaging data associated with catch events.

During Experiment 2, the participants heard short lists of words in a block design paradigm (8-second blocks with eight words each, baseline between blocks 8 seconds). Each run began with a 12-second rest period. Each block contained words from one of the eight concept categories. Experiment 3 was an item-level slow event-related design and was conducted at a different scanning session on the same participants that participated in Exp. 2. The stimuli were eight of the ten words of each category from Experiment 2, except for the concrete object names (see detail above). During each of eight slow event-related runs, the participants heard each word once, in a random order, followed by a 5-second baseline period.

Imaging data for Experiments 2 and 3 were acquired using a Siemens Prisma 3-T scanner with a 20-channel phase-array head coil at the Imaging Center for MRI Research, Peking University. Functional imaging data for Experiment 2 were comprised of four functional runs, each containing 251 continuous whole-brain functional volumes. Functional imaging data for the single-item-level event-related Experiment 3 were comprised of eight functional runs, each containing 209 continuous whole-brain functional volumes. Data were acquired with a simultaneous multi-slice (SMS) sequence supplied by Siemens: slice planes scanned along the rectal gyrus, 64 slices, phase encoding direction from posterior to anterior; 2 mm thickness; 0.2mm gap; multi-band factor = 2; TR = 2000 ms; TE = 30 ms; FA = 90°; matrix size = 112 × 112; FOV = 224 × 224 mm; voxel size = 2 × 2 × 2 mm. T1-weighted anatomical images were acquired for coregistration using a 3D MPRAGE sequence: 192 sagittal slices; 1mm thickness; TR = 2530 ms; TE = 2.98 ms; inversion time = 1100 ms; FA = 7°; FOV = 256 × 224 mm; voxel size = 0.5 × 0.5 × 1 mm, interpolated; matrix size = 512 × 448.

### Data analysis

#### Preprocessing

Imaging data were analyzed using BrainVoyager (BVQX 3.6). Anatomical images were corrected for field inhomogeneities and transformed into Talairach space using 9-parameter affine transformation based on manually identified anatomical landmarks. Functional runs underwent slice time correction, removal of linear trends, and 3D motion correction to the first volume of each run using rigid-body transformation. The first two volumes of each run were discarded to allow for magnetization stabilization. Each run was coregistered with the native-space anatomical image of the same participant using 9-parameter gradient-based alignment, and subsequently warped into Talairach space using the same affine transformation used for warping the anatomical data.

### Whole-brain group-level analysis

**Fig 1:**
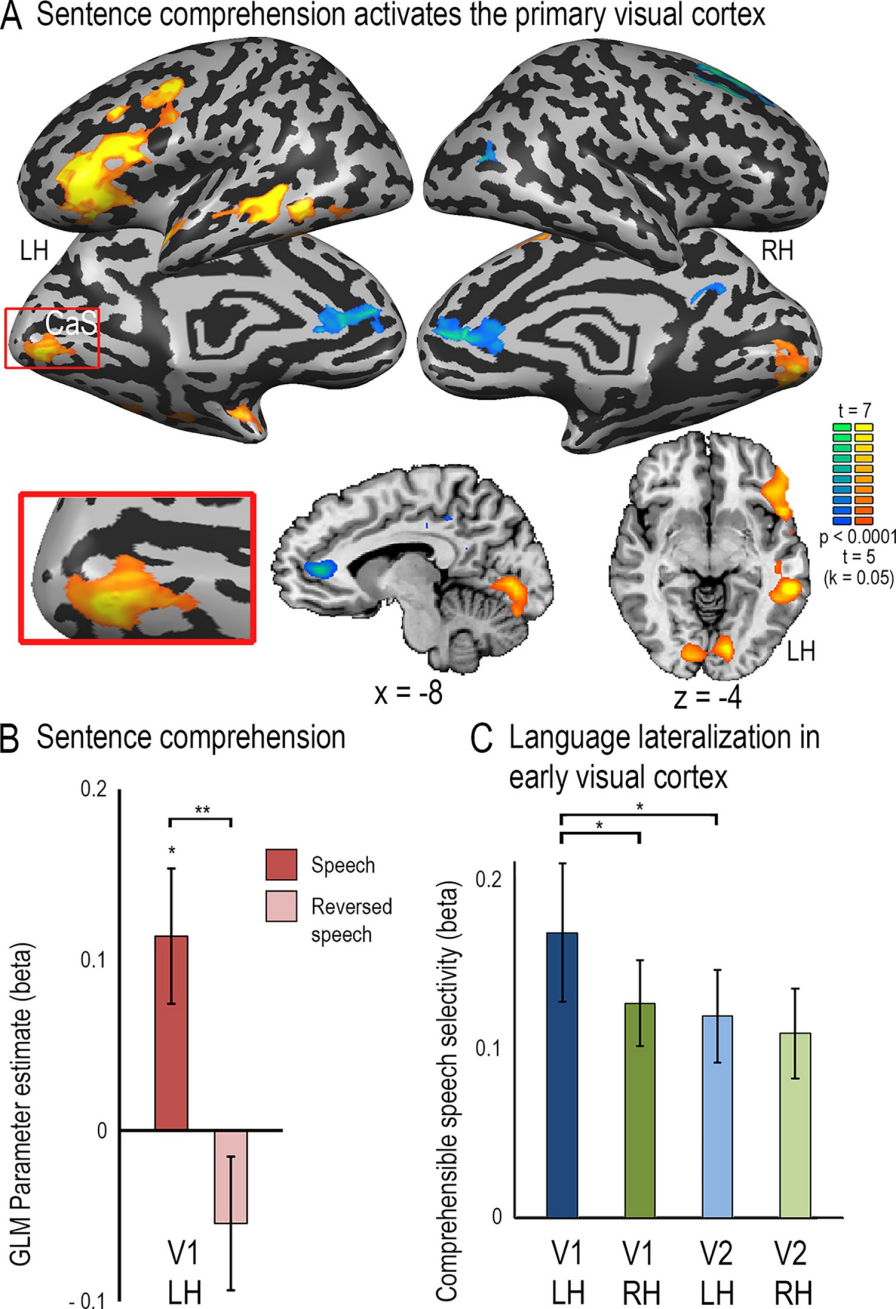
Left primary visual cortex is engaged in spoken language comprehension. **A.** A contrast of comprehensible vs. reversed spoken sentences is shown on brain slices and inflated cortical hemispheres. In addition to the left-lateralized fronto-parieto-temporal language network, significant activation is found in the ventral primary visual cortex. CaS–Calcarine Sulcus. **B.** GLM parameter estimates (betas) were sampled in the left retinotopically-defined primary visual cortex, showing significant activation for comprehensible speech and selectivity for comprehensible vs. reversed speech. Error bars denote standard error of the mean, *p < 0.05, **p < 0.005 FDR corrected. **C.** Selectivity for comprehensible speech (the beta difference between forward and reversed speech) is higher in the left V1 than in right V1, showing slight lateralization for language, and stronger in left V1 compared with left V2 (p < 0.05 FDR corrected for both comparisons). Error bars denote standard error of the mean.

**Fig 2:**
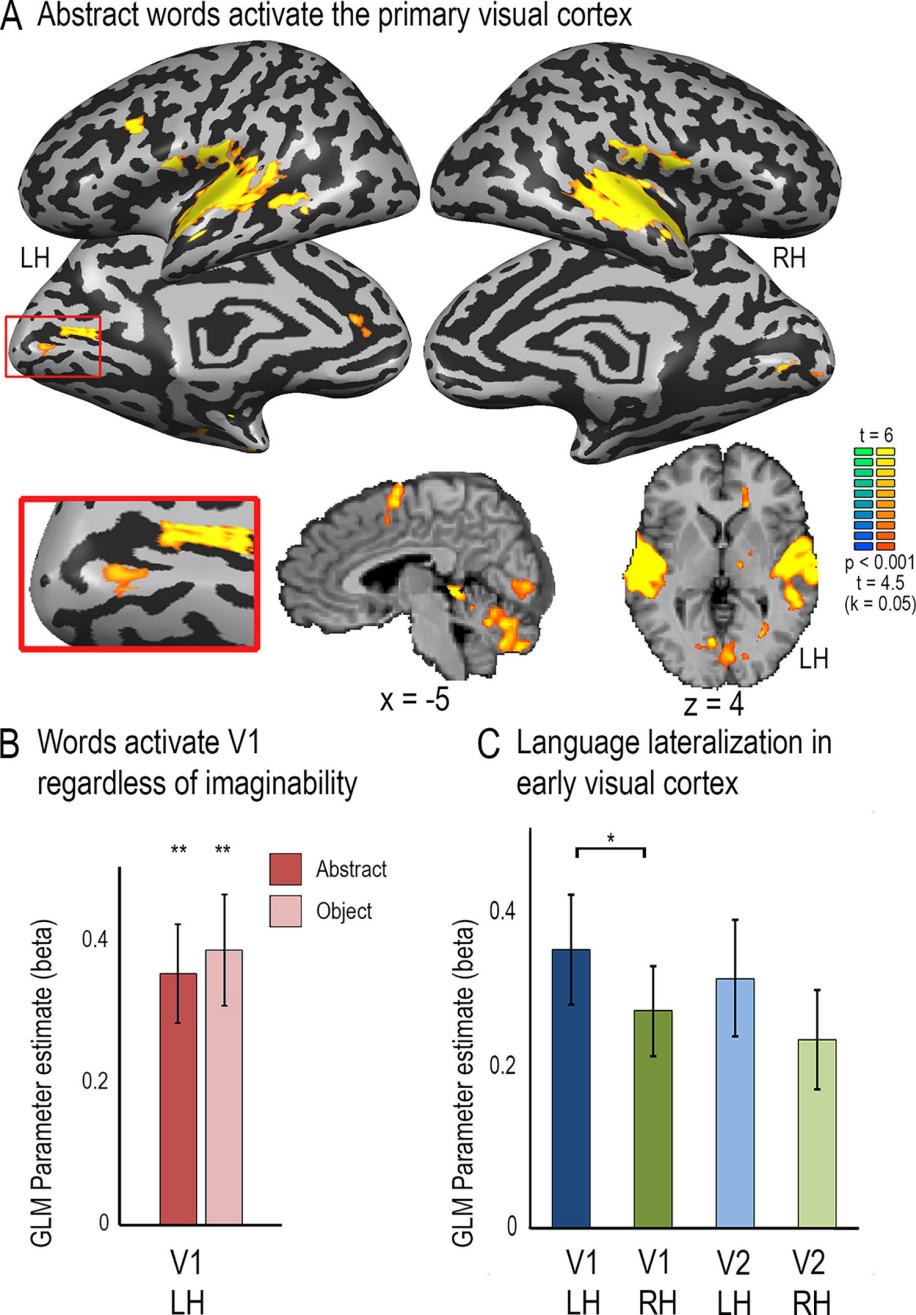
Left primary visual cortex spoken language activation is found for abstract, unimaginable words. **A.** Activation for spoken abstract words as compared to the rest baseline is shown on brain slices and inflated cortical hemispheres. In addition to the auditory cortex and inferior frontal cortex, significant activation is found in the primary visual cortex, despite the inability to visually imagine abstract concepts. CaS–Calcarine Sulcus. **B.** GLM parameter estimates (betas) were sampled in the left retinotopically-defined primary visual cortex, showing significant activation for spoken words, which does not differ between abstract and concrete words. **C.** Activation for abstract words is significantly higher in left than right V1, showing slight lateralization for language. Error bars denote standard error of the mean, *p < 0.05, **p < 0.005 FDR-corrected.

To create group-level activation maps (**Figs 1A and 2A**), we smoothed the Talairach-warped functional data with a 3D Gaussian kernel of 8 mm FWHM and conducted a hierarchical random effects analysis (RFX GLM; [59]). Each experimental condition’s predictor was modeled by convolving the boxcar predictor describing the condition’s time-course with a standard hemodynamic response function (two gamma, peak at 5 s, undershoot peak at 15 s). In addition, the model included nuisance predictors to capture participant- and run-specific effects as well as motion-related effects (using the z-transformed motion estimates generated during preprocessing). During modeling, voxel time courses were normalized using percent signal change transformation and corrected for serial autocorrelations (AR2).

Activation maps contrasting the beta values (GLM parameter estimates) for the different conditions via voxel-wise t-tests were thresholded by applying an uncorrected single-voxel threshold of at least p < 0.001 and running BrainVoyager’s Cluster-Level Statistical Threshold Estimator Plugin to determine a cluster-size threshold corresponding to a corrected threshold k < 0.05.

To control for any attention effects elicited by the onset of sound after periods of rest, GLM analyses for Exp. 1 and Exp. 2 (**Figs 1A and 2A**) were replicated (**S3 and S4 Figs** respectively) with a brief (1TR) condition modelling auditory signal onset at the beginning of each block as a separate predictor.

### Region-of-interest analyses

Regions-of-interest (ROIs) for the primary and secondary visual cortex (V1 and V2, respectively) were defined from an external group localizer [51]. The external retinotopy localizer was acquired in a separate group of 14 normally sighted participants using a standard phase-encoded retinotopic mapping protocol, with eccentricity and polar mapping of ring and wedge stimuli, respectively, to measure visual retinotopic mapping [41, 60–62], delivered during two separate experiments. The stimuli were projected by an LCD projector onto a tangent screen positioned over the subject’s forehead and viewed through a tilted mirror. In the eccentricity experiment, an expanding annulus was presented, expanding from 0 to 34 degrees of the subject’s visual field in 30 s, repeated 10 times. The polar angle experiment presented a wedge with a polar angle of 22.5 degrees that rotated around the fixation point, completing a full cycle in 30 s, repeated 20 times. Both the annulus in the eccentricity experiment and the wedge in the polar angle experiment contained a flickering (6 Hz) radial checkerboard pattern according to standard retinotopic procedures (Engel et al., 1994) for field map mapping. In both cases there was a 30-second period of baseline (fixation) before and after the visual stimulus for baseline. Group phase analysis was conducted on the two experiments as done in other studies [63, 64] resulting in group maps depicting the eccentricity and angle mapping aligned to the Talairach-transformed Colin27 brain. Full experimental detail can be found at [51]. Angle (polar) mapping was used to define the borders of V1 and V2 in both hemispheres, used as a ROI to sample activation for the language conditions in the early visual cortices (**Figs 1B, 1C and 2B, 2C**). V1 was further divided into three portions largely representing foveal, middle, and peripheral visual fields based on the eccentricity mapping (**S2B Fig**).

Beta values (GLM parameter estimates) for each condition were sampled in individuals, and comparisons across conditions within the same ROI (**Figs 1B and 2B**) were computed with a two-tailed paired t-test. Comparisons across areas were computed based on the subtraction of beta values between forward and reversed speech for each individual (**Fig 1C**), and applying a one-tailed paired t-test between regions, under the prediction that language activation would be localized to the left V1, as seen in blindness [23, 24, 26, 35]. Comparably, we used a one-tailed paired t-test between regions to test the hypothesis that abstract word activation vs. baseline would be localized to the left V1 (**Fig 2C**). In addition, to investigate imaginability and arousal as potential contributors to any observed activation, we explored correlations between language activation (GLM parameter estimate, beta values) in the left V1 ROI and imaginability and arousal behavioral ratings of the words presented in Experiment 3, across all 56 words used in the experiment. Lastly, to ensure that the time-course of activation characteristics resemble a genuine neural response, the averaged percent signal change with relation to condition onset was sampled from the left V1 ROI and the standard errors were calculated for each condition and plotted for each time point (**S2A and S2C Fig**).

To statistically correct for these multiple comparisons conducted on our ROI analyses, we report p-values computed using the false-discovery-rate (FDR) approach [65]. Specifically, we corrected for 5 statistical comparisons for the ROI analyses in Experiment 1: (1) V1 forward vs. reverse speech (**Fig 1B**), (2) V1 forward speech vs. silent baseline, (3) left vs. right V1 and (4) Left V1 vs. V2 (**Fig 1C**), and (5) the V1 eccentricity effect (**S2B Fig**). For Experiment 2, we corrected for 4 statistical comparisons: (1) V1 abstract vs. concrete words **(Fig 2B)**, (2) V1 abstract words vs. silent baseline, (3) V1 concrete words vs. silent baseline, and (4) left vs. right V1 (**Fig 2C**).

## Results

### Experiment 1

In contrasting activation by forward and reverse speech in a whole-brain analysis, a typical left-lateralized fronto-temporal “language” network emerged (**Fig 1A**), as identified by numerous neuroimaging studies (for reviews, see [36, 37]). The primary auditory cortex was not significantly activated because the contrasted conditions are matched in low-level auditory information (see similarly [66]), including the sound envelope change rate, which has been suggested to activate V1 [67]. Despite this, the primary visual cortex was significantly activated by forward speech (**S1 Fig**) and more strongly activated by forward than by reverse speech (**Fig 1A**).

This preference was confirmed in region-of-interest (ROI) analyses extracting percent signal change from left and right V1 and V2. Left V1, our primary ROI, showed a significantly stronger response to forward than to reverse speech (**Fig 1B**; paired t-test, t(19) = 4.02, FDR-corrected p = 0.002, d’ = 1.89, one-tailed and displayed a standard stimulus-evoked hemodynamic response for spoken sentences (**S2A Fig**). The response to forward speech was also significant compared to baseline (t(19) = 2.88, FDR-corrected p = 0.012).

A comparison of the forward>reverse speech effect in retinotopically defined V1 in the left and right hemisphere (**Fig 1C**) confirmed the impression from the whole-brain analysis that the activation seemed to be at least somewhat stronger in the left hemisphere (paired t-test, t(19) = 1.98, FDR-corrected p = 0.039, d’ = 0.91). Moreover, the forward>reverse speech effect was slightly weaker in left V2 than in left V1 (paired t-test, t(19) = 2.06, FDR-corrected p = 0.045, d’ = 0.95).

To test whether the V1 activations we observed here might reflect increased attention to the onset of auditory stimulation [68], we repeated the analyses while including a confound predictor modelling a short response to the onset of the conditions. This control analysis replicated the main effects (**S3 Fig**), making simple auditory attention effects an unlikely explanation for the V1 language activations. It is also notable that the whole-brain activations observed here did not include any areas of the fronto-parietal attention network [69–72]) that one might expect to see activated if the forward speech condition elicited a significantly stronger attentional response than the control condition.

### Experiment 2

Could the V1 language activations in Experiment 1 stem from visual imagery, due to the concrete content of the spoken sentences? To explore this possibility, we investigated, in a separate group of sighted adults, whether V1 would show differential activation for abstract (less imaginable) and concrete (easily imaginable) spoken words (e.g., “freedom”, “truth”, “wish” vs. “cup”, “closet”, “computer”). Just as for spoken sentence comprehension (Experiment 1 above), whole-brain activation for listening to blocks of abstract words as compared to inter-block rest interval baseline (Experiment 2; see also [57]) included, in addition to vast activation of the temporal lobe and inferior frontal cortex, also significant localized activation in the calcarine sulcus (**Fig 2A**). Again, activation was stronger in left than in right V1 (**Fig 2C**; paired t-test, t(13) = 2.77, FDR-corrected p = 0.048, d’ = 1.5). Activation time-courses extracted from left V1 resembled a typical hemodynamic response function for both abstract and concrete words (**S2C Fig**). Importantly, left V1 activation did not differ between abstract and concrete words (object names; **Fig 2B**; t(13) = 0.48, FDR-corrected p = 0.975, d’ = 0.27), even though the latter were significantly more imaginable according to behavioral ratings (t(9) = 1074, p < 0.001 uncorrected, significant with correction for multiple comparisons, d’ = 716). As in Exp. 1, modelling the potential attention-arousing effect of the auditory onset at the beginning of each block as a nuisance condition did not affect the main findings (**S4 Fig**).

### Experiment 3

Last, a separate study, an event-related design of spoken words (performed on the same participants and using most of the abstract and easily imaginable words used in Exp. 2; see methods), allowed us to test whether left V1 activation correlated with imaginability and attentional arousal ratings for spoken words of a variety of imaginable and abstract concept types [57]. We computed the correlation between behavioral ratings collected for these words and beta values for each presented word within the left V1 region of interest. No correlation was found between left V1 activation and imaginability ratings (r^2^(54) = 0.003, p = 0.69 FDR corrected) or arousal ratings (r^2^(54) = 0.01, p = 0.90, FDR corrected), although left V1 still showed activation (above baseline) for abstract words (**S5 Fig**). Together, these findings suggest that the observed forward>reverse speech activation in V1 did not result from imagery or attention confounds.

## Discussion

The primary visual cortex is widely thought to be a low-level sensory station devoted to the processing of simple visual features [38–41]. However, there is also increasing evidence implicating the primary visual cortex in aspects of low-level multisensory integration [1–3], and some recent evidence, although still controversial, indicates that it may also receive signals related to higher level non-visual representations, specifically non-visual imagery [14] and working memory [73]. The present results further expand on the known multisensory information reaching V1. We observed activation for spoken sentences in V1 of typically developed individuals, which showed a preference for comprehensible over incomprehensible speech (**Fig 1A, 1B**). Moreover, this activation tended to be stronger in left than right V1 (**Figs 1A, 1C and 2C**), just like the V1 language activation in blindness [24, 26, 34, 35] and in line with the observation of left-lateralization for language activation in the vast majority of adults regardless of handedness [74, 75]. It also tended to be stronger in V1 than in V2, suggesting that it did not emerge from feedback cortico-cortical connectivity from visual language areas via higher retinotopic cortical stations (e.g., V2; **Fig 1C**). The same pattern was evident in response to spoken abstract words in a separate sighted cohort in two separate experiments (**Fig 2 and S5 Fig**). Together these findings suggest that left-lateralized primary visual cortex responds to spoken language information in typically developed sighted adults. These findings reflect on several key issues regarding the multisensory properties of primary visual cortex, the developmental origins of reorganization in the blind brain, and the nature of brain plasticity itself.

Before we discuss these implications, we must first address why our study is the first to highlight V1 language activation in sighted individuals despite the large number of functional neuroimaging studies that have investigated language activation in the typically-developed population. One likely reason is that V1 activation is small relative to that in other regions of the frontotemporal language network, both regarding peak signal change and regarding spatial extent. Thus, depending on the statistical power of the experiment and applied thresholds, V1 activation may not be apparent in all functional neuroimaging studies contrasting comprehensible speech with silence, non-speech stimuli, or incomprehensible speech stimuli. However, it is apparent in some [76–84]. In studies directly looking for early visual cortex recruitment in blindness, which included sighted participants as a control group, there is at times mention of responses in the sighted [24, 85], but often, possibly due to statistical analysis and power or to the specific contrasts used, significant activation in the sighted is either not found [23, 28, 32, 86, 87], or is (accurately) reported as smaller than that of the blind group without being tested for significance in and of itself [26]. In summary, while our experiments are not the first to show V1 activation in language tasks in sighted adults, our focus on this activation and examination of its properties with relevant controls allows us to interpret it as a meaningful response to spoken language.

To do so, we must address whether our findings could be explained by confounds such as differences in visual stimuli, visual imagery or increased attention to speech which in themselves can generate responses in V1 [88–91], as has been suggested for several other studies observing early visual cortex activation by language stimuli [76, 77, 79, 82]. Visual stimulation was matched and essentially absent throughout our experiments: In Experiment 1, participants were instructed to rest their eyes on a fixation cross throughout, matching visual stimulation during forward and reverse speech blocks; and during Experiments 2 and 3, participants listened with their eyes closed and blindfolded. This rules out unmatched visual stimulation between the conditions as a potential explanation for the observed V1 activation.

There are also several reasons to argue that global attention is an unlikely explanation for the V1 activation observed here. First, when attention to sounds activates V1, it likely serves a role in spatial attention orientation, found more strongly in peripheral V1 [13, 91, 92] and stems from direct and indirect anatomical connectivity between auditory cortices and mostly (though not only) *peripheral* retinotopic locations of V1 found in primates and humans [93–99]. In contrast, the activation pattern in comparing forward and reversed speech in our study was not peripherally localized (**S2B Fig**; ANOVA for an eccentricity effect in left V1 F(2,143) = 0.01, p = 0.99). Second, no activation was observed in the fronto-parietal attention network (typically bilateral or right-lateralized; [69–72]) in our whole-brain analysis, which would be expected if there were significant attention differences between the conditions (either due to larger top-down attention allocated to processing the comprehensible stimulus, or to larger effort to selectively attend to that stimulus over the scanner noise). Moreover, including an additional nuisance regressor to capture attention effects associated with sound onset did not abolish the V1 response in either block-design experiment (**S3 and S4 Figs**). Lastly, activation in left V1 was not correlated with the arousal ratings of the heard words in Experiment 3. All this makes it unlikely that attention is the primary driver for the V1 responses we observed.

Similarly, it does not appear to stem from visual imagery. Visual imagery may activate and its content can be decoded from the primary visual cortex [89, 100–106]. However, imagery responses are stronger in association rather than primary cortex [107, 108] and are typically bilateral [104, 108, 109]. When V1 activation is reported for visual imagery, it is usually associated with explicit imagery of high-resolution detail of images [89, 110, 111]. In contrast, our sentence comprehension task did not require explicit imagery or attention to visual detail, activation was slightly stronger in V1 than V2 (**Fig 1A, 1C**), and elicited no activation or deactivation as compared to rest in other retinotopic and association visual areas (**S1 Fig**). Moreover, we observed the same localized V1 activation in a whole-brain analysis for abstract words (**Fig 2A and 2C**), and it was no weaker for abstract than concrete words (**Fig 2B**) even though the former were rated as significantly less imaginable. The V1 response also was not correlated with imaginability ratings (Experiment 3). This pattern of results argues against visual imagery as an explanation for the observed V1 activation.

Having ruled out these potential confounds to the best of our ability, we lean towards interpreting the V1 responses to forward>reverse speech (Experiment 1) and to spoken words of varying imaginability (Experiments 2 and 3) as activation driven by spoken language. However, our results do not reveal which aspect(s) of spoken language processing are contributing to this activation. Our reversed speech condition controls for several aspects, such as the overall spectral and temporal envelope of the forward speech stimuli, which may in itself drive V1 activation [67, 68]. Nonetheless, it does differ from the forward speech condition in several ways beyond the lack of linguistic information and meaning. One difference between the forward and reversed speech is that reverse speech contains sounds that are not usually (and in some cases cannot be) produced by the human vocal apparatus. If V1 response are driven by the visual or cross-modal associations of spoken language’s common speech sounds [112], this could also form a difference in Exp. 1. Further, although reversed speech is well matched to forward speech for long-term spectrotemporal characteristics, including the overall spectral and temporal envelopes, time-reversal disrupts local spectrotemporal patterns. Yet, it appears unlikely that V1 would be more sensitive to these features than A1, which does not show differences between reversed and forward speech (**Fig 1A**). The most obvious difference between forward and reverse speech remains the presence of and attention to linguistic information and meaning. This difference is what the present study shares with the other studies that also found V1 language activation in sighted adults [76–84]. All these studies were conducted using different designs and stimuli, and while each individual one may have potential confounds, the most parsimonious explanation for the V1 activation common to all of them is what all studies’ contrasts have in common with each other: linguistic and semantic processing.

How might linguistic information reach V1 and what role could it play? Primary sensory cortices receive information from multiple cortical and subcortical stations. Specifically, beyond thalamic LGN and pulvinar projections, primary visual cortex receives input from auditory cortices, parietal cortex and other regions including frontal cortex in primates and other mammals [94–96, 98, 113–119]. These feedback pathways [119, 120] allow for multisensory integration even in V1 [1–3, 9, 121], along with integration of reward value information [122, 123]. Relevant non-visual information can affect early visual cortex excitability [4], and interact with visual EEG alpha wave phase [6, 8, 124]. Many studies have shown early visual cortex responses to, modulation of, or causal involvement in the processing of simple sound stimuli (e.g. [4–10]; see [1] for a review), often based on their spatial location or temporal synchrony [11–13]. However, it is less clear whether early visual cortex receives information pertaining to high-level auditory information such as object category, congruence, or language. Vetter and colleagues [14, 125] showed that V1 signal patterns can discriminate the categorical content of sounds both in sighted and congenitally blind individuals, but others did not find such high-level modulations [15–22]. Moreover, recent studies showed that the primary visual cortex may represent information pertaining to the temporal envelope for complex sounds [67, 68], suggesting that the successful discrimination of sound category from V1 activation patterns could have been based on mid-level properties of the sounds rather than high-level semantic category information. However, in our experiment, the sound temporal envelope modulation frequency was maintained in the reversed speech control condition, which demonstrates that the preference of V1 for comprehensible speech goes beyond sound envelope.

Although the representational content of language responses in V1 will need to be further addressed by multivariate analyses, our results provide additional evidence for semantic processing of auditory stimuli in V1. Theoretically, these cross-modal and higher-level inputs to V1 could play a role in predictive coding, whereby predictions of future states and inputs enables visual cortex to anticipate coming events [119, 126–128] and allows for efficient coding and adapting to the everchanging environment [3, 117, 119]. How language comprehension fits into this framework is uncertain. Language input into V1 may allow integrating contextual information that enables visual cortex to anticipate coming events [119, 126, 127]. Alternatively, it may play a simpler role in alerting spatial or overall attention [13, 129], without conveying specific content. What we tentatively interpret as V1 language activation may even be epiphenomenal altogether; our data do not speak directly to these alternative explanations, which will need to be addressed in future work. Importantly, accounts of predictive use of speech information would have to reconcile the level of representation of incoming high-level inputs with the spatial and low-level nature of V1 (e.g., [129]), such that these types of information can be integrated in a meaningful way.

While our data cannot speak to the functional role of V1 language activation in the sighted, the resemblance of the V1 activation observed here to that seen in people born blind is intriguing. In people born blind early retinotopic visual cortices, including V1, are activated by high-level cognitive tasks such as language, verbal memory and executive function [23–28, 31, 32, 34, 86, 130–132]. Stimulating primary visual cortex affects Braille reading [133] and verb generation [33] suggesting that, at least in people born blind, this activation may indeed contribute to language processing. The fact that similar (albeit weaker) V1 language activation can be seen in the sighted brain suggests that such activation in the blind may not require massive changes in brain organization. This is particularly important because despite evidence suggesting increased functional connectivity between early visual cortex and the inferior frontal lobe in the blind [51, 134–138], no anatomical evidence for such large changes has been identified to date. Rather, differences in anatomical connectivity between sighted and early blind individuals, although evident in some animal models [139–142], in humans appear to be limited in scope, mostly to the deterioration of the visual pathways in the blind [42–45]. Responses to language in V1 in the sighted indicate that even major functional reorganization (e.g. functional pluripotency [143]) may not be needed either. Rather, our data suggest a more conservative explanation of V1’s language recruitment in blindness: little reorganization of V1 structure or function (perhaps in the shape of unmasking [144], and additional local changes) is required to support language recruitment of deprived cortex because it also recruits non-deprived cortex, albeit to a lower extent. This is in line with similar explanations that have already been proposed for non-visual responses in the visual cortex in blindness for other perceptual [48, 145–147] and cognitive [148, 149] domains. Importantly, no comparable explanation was possible for language recruitment in V1, given that V1 language responses were not reported in the sighted brain. Our evidence here closes this gap, and reconciles the seemingly inordinate plasticity for language in people born blind with current views of connectivity-driven functional brain organization [46–49, 150]. Thus, we contribute to a unifying explanatory framework for findings in the primary and association cortices in the blind, based on extant non-visual functions of the visual cortex.

In summary, our findings expand on the known non-visual, cognitive inputs to the primary visual cortex and suggest its modulation also by cognitive inputs even in the sighted brain, possibly as part of a predictive coding mechanism guiding visual perception. These findings also provide evidence that language-driven visual cortex activation in the blind can be explained without proposing drastic changes to cortical tissue connectivity or function. This suggests that human cortical plasticity is still limited by innate anatomical structures and functional characteristics and is not unconstrained even following extreme changes in early experience.

## Supporting information

S1 Fig Left primary visual cortex is activated by spoken language. A contrast of comprehensible spoken sentences as compared to baseline is shown on brain slices and inflated cortical hemispheres. In addition to the auditory cortex and left-lateralized fronto-parieto-temporal language network, significant activation is found in the primary visual cortex, accompanied by deactivation of other parts of early visual cortex. CaS–Calcarine Sulcus.
(DOCX)

S2 Fig Activation for speech comprehension in primary visual cortex. **(A)** Time course of activation from Experiment 1 was sampled from retinotopic left V1, showing typical BOLD-shaped response in V1 for speech, which is higher for forward as compared to reversed speech. (**B**) GLM parameter estimates (betas) were sampled in the left retinotopically-defined primary visual cortex divided based on eccentricity, showing that the activation for forward speech does not differ between foveal, middle and peripheral-representing V1 sections. (**C**) Time course of activation from Experiment 2 was sampled from retinotopic left V1, showing typical BOLD-shaped response in V1 for abstract and concrete words.
(DOCX)

S3 Fig Left primary visual cortex is engaged in spoken language comprehension when including a “block onset” predictor to control for bottom-up attention effects. This figure is provided for comparison with Fig 1. The underlying analyses are the same except for the inclusion of an additional nuisance predictor in the GLM to capture the bottom-up attention effects that might occur at the beginning of each block, at the onset of auditory stimulation. Even after including this additional predictor, a left-lateralized fronto-temporal language network is clearly evident (A), left V1 activation is significantly stronger for comprehensible forward than incomprehensible reverse speech (B), and the forward>reverse effect is slightly stronger in left V1 compared to right V1 and left V2 (C). **p < 0 .01, *p < 0.05 FDR corrected.
(DOCX)

S4 Fig Left primary visual cortex spoken language activation is found for abstract, unimaginable words when including a “block onset” predictor to control for bottom-up attention effects This figure is provided for comparison with Fig 2. The underlying analyses are the same except for inclusion of an additional nuisance predictor in the GLM to capture the bottom-up attention effects that might occur at the beginning of each block, at the onset of auditory stimulation. Even after including this additional predictor, activation of the primary visual cortex for abstract words is clearly evident (A), with no difference in response between abstract and concrete (object name) words (B; p = 0.94), and the response to abstract words is stronger in left V1 compared to right V1 (C). *p < 0.05 FDR corrected.
(DOCX)

S5 Fig Left primary visual cortex spoken language activation is replicated for abstract words in Experiment 3. Activation for spoken abstract words in Experiment 3 as compared to the rest baseline is shown on brain slices and inflated cortical hemispheres. In addition to the auditory cortex and inferior frontal cortex, significant activation is found in the left primary visual cortex, replicating the findings of Experiments 1 and 2. CaS–Calcarine Sulcus.
(DOCX)

S1 Audio Example of a correct spoken sentence as used in Experiment 1. (WAV)

S2 Audio Example of an incorrect spoken sentence as used in Experiment 1. (WAV)

S3 Audio S1 Audio played in reverse and thus rendered incomprehensible. (WAV)

S4 Audio S2 Audio played in reverse and thus rendered incomprehensible. (WAV)
