# Supplementary figures and images for: Spoken language processing activates the primary visual cortex
Source: PLoS One. 2023 Aug 11;18(8):e0289671. doi: 10.1371/journal.pone.0289671 (PMC10420367; doi:10.1371/journal.pone.0289671)

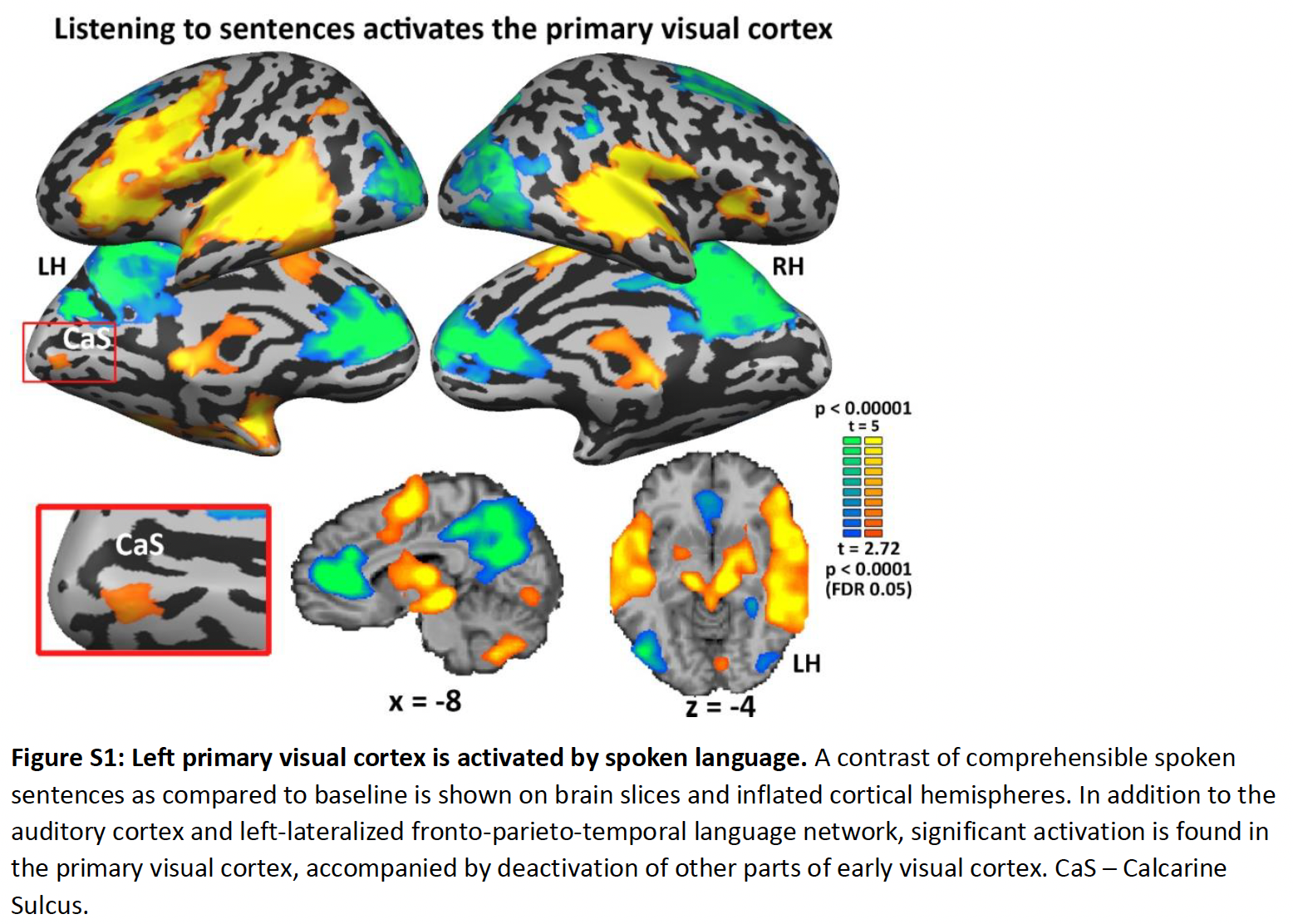

Supplement: S1 Fig — A contrast of comprehensible spoken sentences as compared to baseline is shown on brain slices and inflated cortical hemispheres. In addition to the auditory cortex and left-lateralized fronto-parieto-temporal language network, significant activation is found in the primary visual cortex, accompanied by deactivation of other parts of early visual cortex. CaS–Calcarine Sulcus. (DOCX) [file pone.0289671.s001.docx]

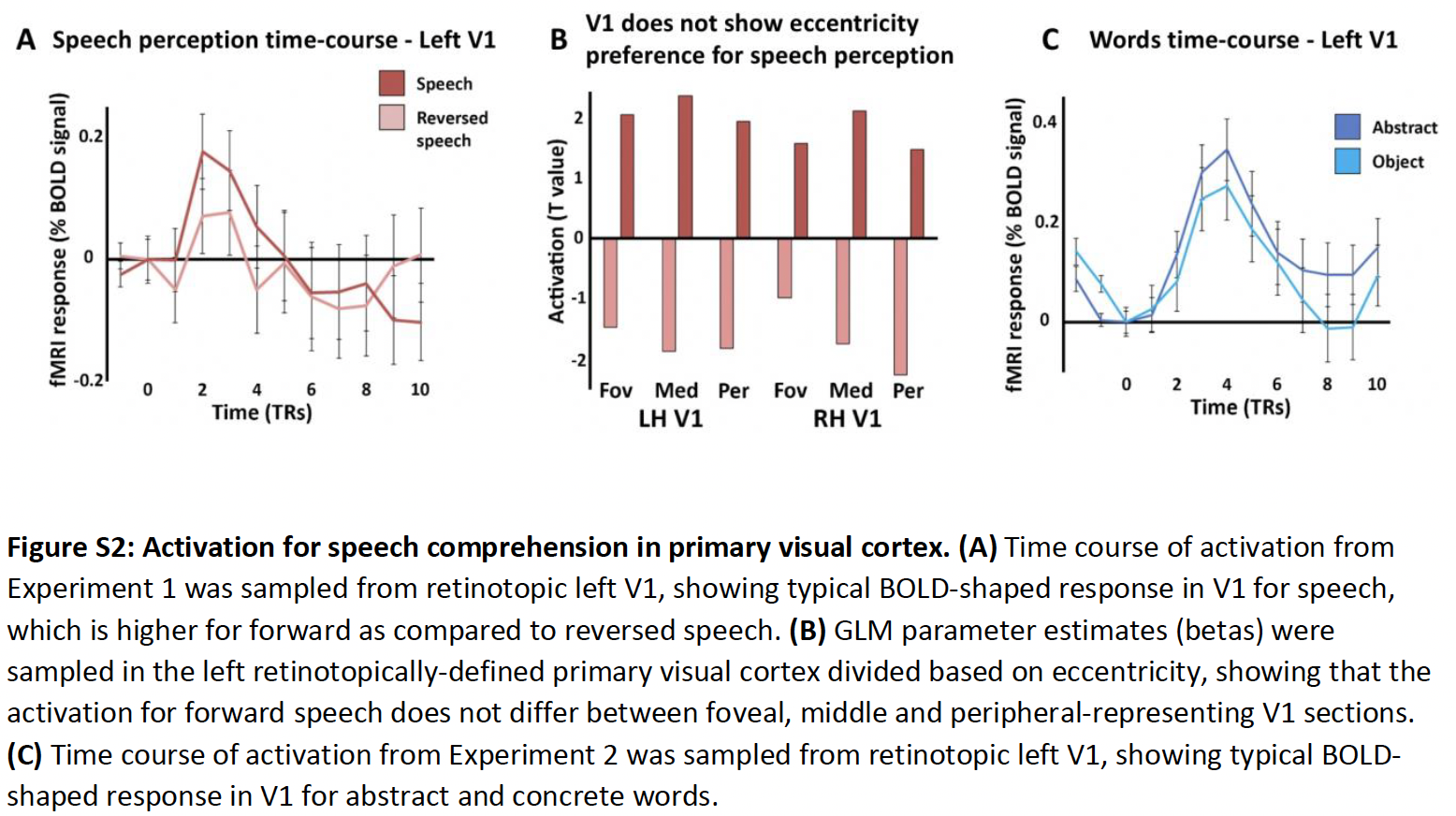

Supplement: S2 Fig — (A) Time course of activation from Experiment 1 was sampled from retinotopic left V1, showing typical BOLD-shaped response in V1 for speech, which is higher for forward as compared to reversed speech. (B) GLM parameter estimates (betas) were sampled in the left retinotopically-defined primary visual cortex divided based on eccentricity, showing that the activation for forward speech does not differ between foveal, middle and peripheral-representing V1 sections. (C) Time course of activation from Experiment 2 was sampled from retinotopic left V1, showing typical BOLD-shaped response in V1 for abstract and concrete words. (DOCX) [file pone.0289671.s002.docx]

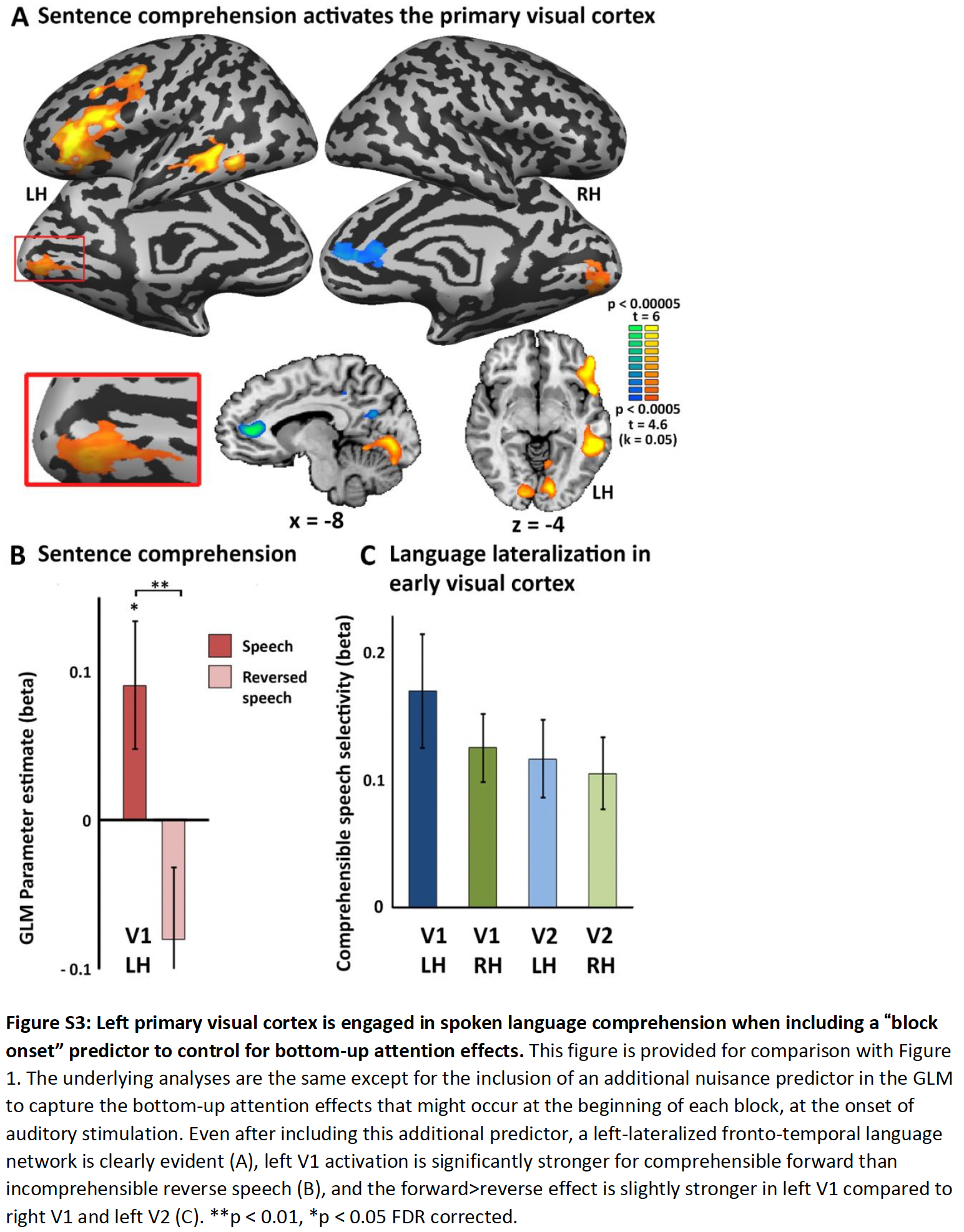

Supplement: S3 Fig — This figure is provided for comparison with Fig 1. The underlying analyses are the same except for the inclusion of an additional nuisance predictor in the GLM to capture the bottom-up attention effects that might occur at the beginning of each block, at the onset of auditory stimulation. Even after including this additional predictor, a left-lateralized fronto-temporal language network is clearly evident (A), left V1 activation is significantly stronger for comprehensible forward than incomprehensible reverse speech (B), and the forward>reverse effect is slightly stronger in left V1 compared to right V1 and left V2 (C). **p < 0 .01, *p < 0.05 FDR corrected. (DOCX) [file pone.0289671.s003.docx]

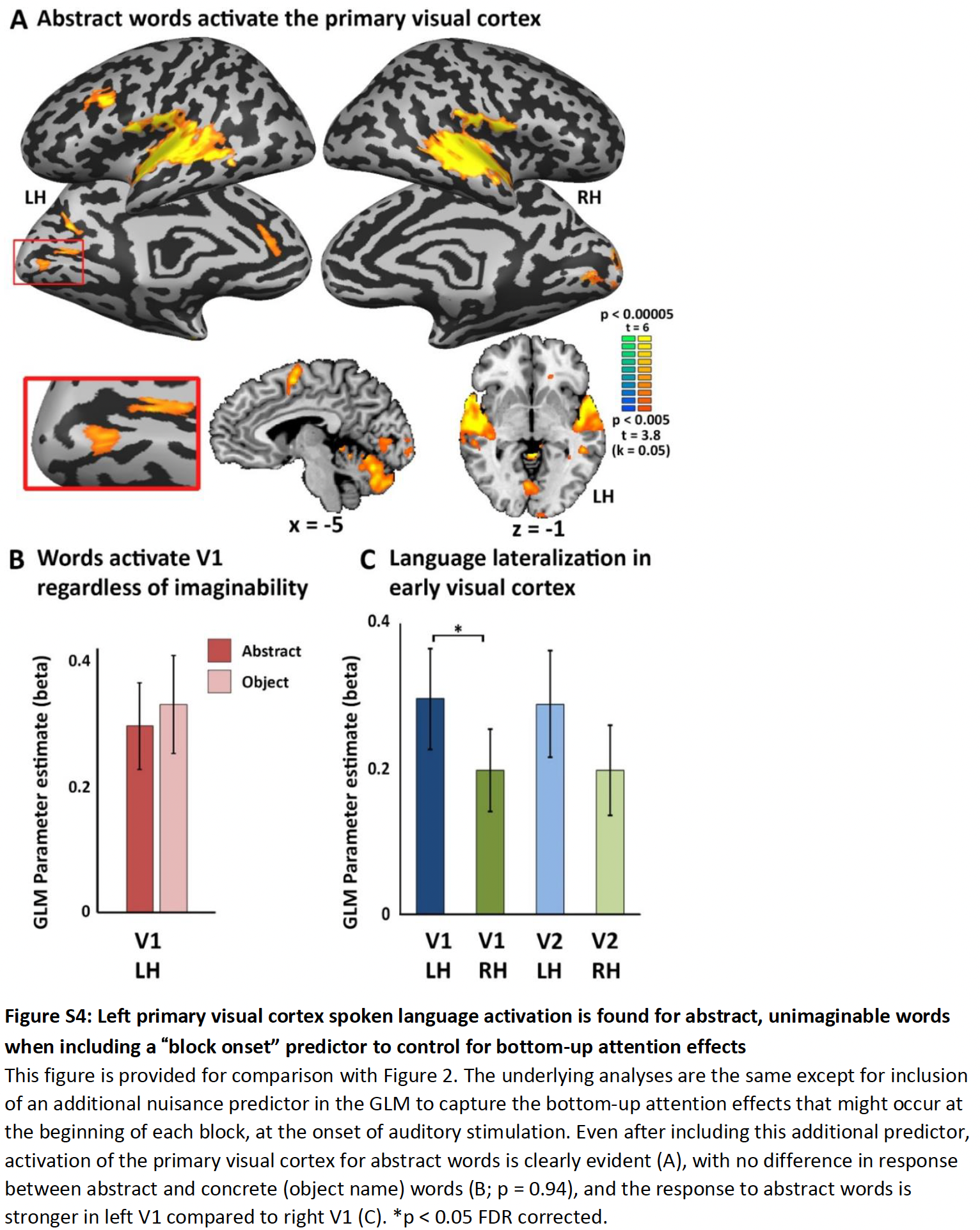

Supplement: S4 Fig — The underlying analyses are the same except for inclusion of an additional nuisance predictor in the GLM to capture the bottom-up attention effects that might occur at the beginning of each block, at the onset of auditory stimulation. Even after including this additional predictor, activation of the primary visual cortex for abstract words is clearly evident (A), with no difference in response between abstract and concrete (object name) words (B; p = 0.94), and the response to abstract words is stronger in left V1 compared to right V1 (C). *p < 0.05 FDR corrected. (DOCX) [file pone.0289671.s004.docx]

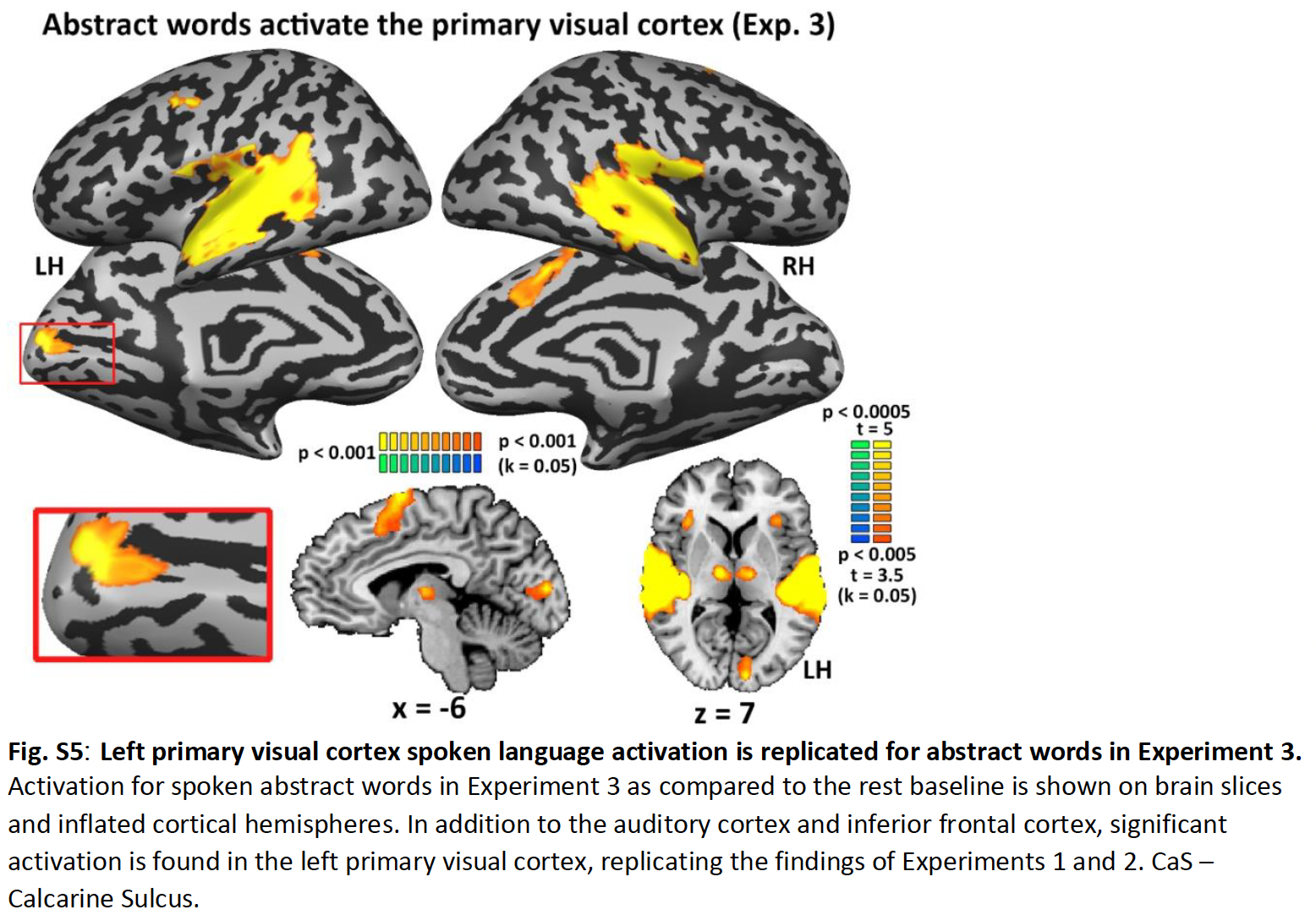

Supplement: S5 Fig — Activation for spoken abstract words in Experiment 3 as compared to the rest baseline is shown on brain slices and inflated cortical hemispheres. In addition to the auditory cortex and inferior frontal cortex, significant activation is found in the left primary visual cortex, replicating the findings of Experiments 1 and 2. CaS–Calcarine Sulcus. (DOCX) [file pone.0289671.s005.docx]
